# Bidding the CpG island goodbye

**DOI:** 10.7554/eLife.00593

**Published:** 2013-02-26

**Authors:** John M Greally

**Affiliations:** 1 **John M Greally** is at the Department of Genetics, Albert Einstein College of Medicine, New York, United Statesjohn.greally@einstein.yu.edu

**Keywords:** CpG islands, DNA methylation, Epigenetics, Chromatin, Evolutionary conservation, Chicken, Human, Mouse

## Abstract

Experiments on seven vertebrates suggest that identifying the locations of islands of non-methylated DNA provides more insights into evolutionarily-conserved epigenetic regulatory elements than studies of CpG islands.

**Related research article** Long HK, Sims D, Heger A, Blackledge NP, Kutter C, Wright ML, Grützner F, Odom DT, Patient R, Ponting CP, Klose RJ. 2013. Epigenetic conservation at gene regulatory elements revealed by non-methylated DNA profiling in seven vertebrates. *eLife* **2**:e00348. doi: 10.7554/eLife.00348

**Image** Percentage of non-methylated islands of DNA conserved among seven vertebrates

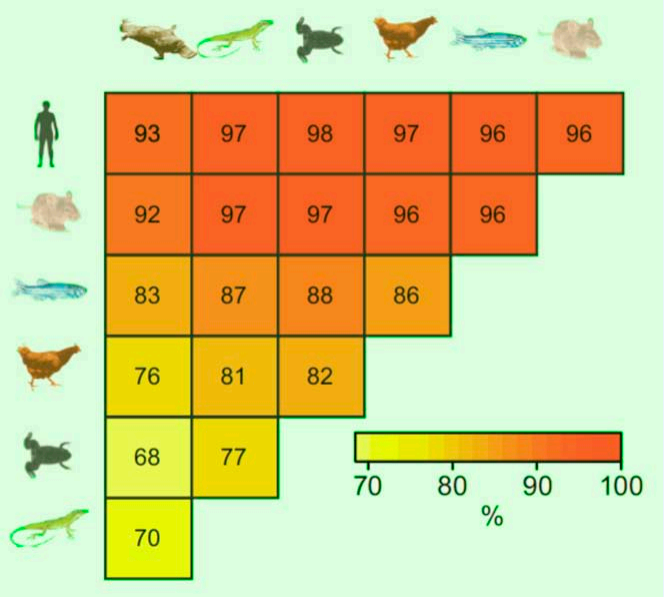

It is now almost 26 years since the CpG island—a stretch of DNA with a larger than expected proportion of cytosine followed by guanine bases—was first defined, based on an analysis of the relative proportions of the four bases in the then limited amount of human sequence information available (Gardiner-Garden and Frommer, 1987). At the time, these islands of CpG dinucleotides were presumed to be the location of *cis*-regulatory elements (regions of DNA that regulate the expression of nearby genes) and, in particular, to be the location of gene promoters (regions of DNA that initiate the transcription of genes).

During the past quarter century, we have sequenced numerous whole genomes from a wide range of species, and have witnessed the development of powerful techniques for identifying *cis*-regulators throughout these whole genomes, yet we still persist with the concept of the CpG island when we annotate those parts of the genome that do not code for proteins. Frequently ignored is the fact that the annotation only works if we exclude the substantial proportion of the genome that is repetitive DNA, mostly the remnants of self-replicating virus-like elements that have all of the sequence characteristics of the CpG island but are rarely found to be regulatory elements (Glass et al., 2007). A defining feature of CpG islands is that they tend to escape DNA methylation (the addition of a methyl group to cytosine), whereas cytosines in the genome as a whole, and in repetitive DNA in particular, tend to be heavily methylated (Yoder et al., 1997). The question that emerges is whether the CpG island annotation merely acts as a surrogate for an absence of DNA methylation, which is much more relevant when we are searching for *cis*-regulators in the genome.

Now, in *eLife*, Robert Klose, Chris Ponting and colleagues at Oxford University, Cancer Research UK and the University of Adelaide—including Hannah Long and David Sims of Oxford as joint first authors—highlight the weakness of the CpG island annotation in an innovative way. They report that when they looked for loci that escape DNA methylation in a set of non-human genomes, they found the CpG island annotation to be very poorly associated with these unmethylated loci (Long et al., 2013). They used a technique called biotinylated CxxC affinity purification (Bio-CAP), followed by massively parallel sequencing, to identify islands of non-methylated DNA in seven highly divergent vertebrate species, ranging from fish to humans.

The Bio-CAP approach takes advantage of the fact that CxxC protein domains (where x is an amino acid other than cysteine) bind preferentially to CpG dinucleotides that are not methylated (Voo et al., 2000). Long, Sims and co-workers found that the base composition of the non-methylated islands in the different species varied substantially. Moreover, the non-methylated islands were conserved more between the species than the CpG islands were, which suggests that they are more biologically meaningful. The results also demonstrate that the CpG island annotation performs especially poorly in non-human species.

The Bio-CAP approach is likely to have its own limitations: the CxxC domain is more likely to capture and enrich loci with multiple unmethylated CpG dinucleotides on the same fragment of DNA, so longer stretches of unmethylated sequence, especially if they are rich in CpG dinucleotides, are going to be more readily identified. The use of 51 base pair single-end reads in the Bio-CAP approach also makes it less likely that non-methylated islands in repetitive DNA (where it is more difficult to map such short reads) will be identified, should they happen to exist. However, as a survey technique, the Bio-CAP approach has many strengths. It should also be recognized that shotgun bisulphite sequencing, the gold standard for DNA methylation studies, does not comprehensively test every cytosine in the genome (Harris et al., 2010), strengthening the justification for survey techniques in the short term until a better genome-wide approach is developed.

The use of mixed cell types in the tissues studied might also influence the results, by tending to enrich those non-methylated islands that are found in many different types of cells. However, despite this possibility, Long, Sims and co-workers were able to compare cells taken from the liver and testes and identify non-methylated islands that were specific to each tissue type. The tissue-specific islands were shorter and contained fewer CpG dinucleotides than those found in both types of tissue, a finding that is reminiscent of work at Stanford that identified two classes of gene promoters—one with high levels of CpG dinucleotides and one with lower levels (Saxonov et al., 2006).

So where does this new insight about non-methylated islands leave us? Base composition has served us well for over a quarter of a century in defining the candidate *cis*-regulatory elements we call CpG islands, but we are now in a different era in which functional elements can be annotated at high resolution based on molecular assays in individual cell types. At first these annotations were generated by large collaborations—such as the ENCODE collaboration (Dunham et al., 2012), the modENCODE collaboration (Celniker et al., 2009), and the Roadmap in Epigenomics (Bernstein et al., 2010)—but it is becoming increasingly feasible for individual investigators to generate such annotations. This has enormous potential value in allowing us to understand the information located at non-protein coding sequences in the genome. Moreover, as Long, Sims and colleagues clearly demonstrate, the ability to do this is a prerequisite for performing comparative studies between species.

The problem that will arise in a new era of functional annotations will be that of community standards—most people have tended to agree what defines a CpG island, but definitions of features based on identifying unmethylated DNA are likely to be more contentious. For example, is there a minimum size for these features? If a single CpG dinucleotide remains unmethylated in all the cell types tested, surely it should be considered as a potentially significant locus? And if a locus is partially unmethylated on a consistent basis, how unmethylated does it have to be to be a candidate regulatory element? Is conservation of DNA methylation patterns the best way to identify candidates for regulatory elements, or are there other ways?

Notwithstanding these concerns, the work described by Long, Sims and colleagues represents the kind of bold and empirically-based approach that we need to develop for every cell type from every research organism. In parallel, the CpG island annotation on every genome browser should now come with a user warning, especially for non-human genomes: after 26 years of service, the CpG island should be allowed to retire with honour.
